# Addition of milk fat globule membrane-enriched supplement to a high-fat meal attenuates insulin secretion and induction of soluble epoxide hydrolase gene expression in the postprandial state in overweight and obese subjects

**DOI:** 10.1017/jns.2019.11

**Published:** 2019-04-26

**Authors:** Elizabeth Beals, S. G. Kamita, R. Sacchi, E. Demmer, N. Rivera, T. S. Rogers-Soeder, E. R. Gertz, M. D. Van Loan, J. B. German, B. D. Hammock, J. T. Smilowitz, A. M. Zivkovic

**Affiliations:** 1 Department of Nutrition, University of California, Davis, CA, USA; 2 Department of Entomology, University of California, Davis, CA, USA; 3 US Department of Agriculture/Agricultural Research Service Western Human Nutrition Research Center, Davis, CA, USA; 4 Foods for Health Institute, University of California, Davis, CA, USA; 5 Department of Food Science & Technology, University of California, Davis, CA, USA

**Keywords:** Milk fat globule membrane, Postprandial inflammation, Saturated fat, Cytokines, Metabolic syndrome, Overweight, Inflammatory markers, ARA, arachidonic acid, CD14, cluster of differentiation 14, Chol:HDL, cholesterol:HDL-cholesterol ratio, CRP, C-reactive protein, EPHX2, soluble epoxide hydrolase, iAUC, incremental AUC, LBP, lipopolysaccharide binding protein, LPS, lipopolysaccharide, LTBR, lymphotoxin *β* receptor, MetS, metabolic syndrome, MFGM, milk fat globule membrane, SAA, serum amyloid A, sEH, soluble epoxide hydrolase, T2DM, type 2 diabetes mellitus, WC, whipping cream

## Abstract

CVD and associated metabolic diseases are linked to chronic inflammation, which can be modified by diet. The objective of the present study was to determine whether there is a difference in inflammatory markers, blood metabolic and lipid panels and lymphocyte gene expression in response to a high-fat dairy food challenge with or without milk fat globule membrane (MFGM). Participants consumed a dairy product-based meal containing whipping cream (WC) high in saturated fat with or without the addition of MFGM, following a 12 h fasting blood draw. Inflammatory markers including IL-6 and C-reactive protein, lipid and metabolic panels and lymphocyte gene expression fold changes were measured using multiplex assays, clinical laboratory services and TaqMan real-time RT-PCR, respectively. Fold changes in gene expression were determined using the Pfaffl method. Response variables were converted into incremental AUC, tested for differences, and corrected for multiple comparisons. The postprandial insulin response was significantly lower following the meal containing MFGM (*P* < 0·01). The gene encoding soluble epoxide hydrolase (*EPHX2*) was shown to be more up-regulated in the absence of MFGM (*P* = 0·009). Secondary analyses showed that participants with higher baseline cholesterol:HDL-cholesterol ratio (Chol:HDL) had a greater reduction in gene expression of cluster of differentiation 14 (*CD14*) and lymphotoxin *β* receptor (*LTBR*) with the WC+MFGM meal. The protein and lipid composition of MFGM is thought to be anti-inflammatory. These exploratory analyses suggest that addition of MFGM to a high-saturated fat meal modifies postprandial insulin response and offers a protective role for those individuals with higher baseline Chol:HDL.

CVD and type 2 diabetes mellitus (T2DM) are both linked with chronic inflammation^(^^1^^)^. Diet plays a major role in influencing inflammation at the vascular wall and in peripheral tissues, where atherosclerosis and insulin resistance can occur^(^^2^^,^^3^^)^. Obesity is another major contributor to chronic inflammation and increases an individual's risk for hypertension, dyslipidaemia, hyperglycaemia and T2DM. Together, these co-morbidities are termed the metabolic syndrome (MetS), and are mediated by inflammatory processes in the body^(^^4^^–^^7^^)^. It has been estimated that 35 % of adults have traits of the MetS, a figure that jumps to 50 % in adults over the age of 60 years^(^^8^^)^. The MetS increases the risk of T2DM, which is associated with increased risk of retinopathy, infection and peripheral neuropathy, which can result in amputations and blindness^(^^7^^,^^9^^–^^12^^)^.

The magnitude of the postprandial (or immediately following a meal) inflammatory response plays a role in the progression of CVD and exacerbates the risk of developing the MetS in individuals with existing chronic inflammation^(^^13^^)^. In Western societies, most of the day is spent in the postprandial period, with only a few hours in the early morning spent in the fasted state^(^^14^^–^^16^^)^. Risk for chronic metabolic disease may be more apparent by looking at the inflammatory response following a meal, as opposed to looking at fasting markers of inflammation^(^^14^^)^. Furthermore, meal composition is an important determinant of postprandial macronutrient metabolism and inflammation. Saturated fat from any dietary source, including dairy products, was once thought to be a major contributor to CVD risk. A few reviews summarising numerous randomised controlled trials and epidemiological studies showed that while consumption of dairy products may improve certain clinical biomarkers that are associated with CVD risk, there is not enough evidence to state whether consumption of dairy products is neutral or beneficial to overall CVD risk^(^^17^^–^^19^^)^. Additionally, differences exist in clinical end points for high-fat, low-fat, fermented and total dairy products^(^^18^^,^^20^^,^^21^^)^. The fatty acid composition and other bioactive molecules in conjunction with the saturated fat may alter the overall physiological response. Milk fat globule membrane (MFGM) is a component of dairy foods found in the lipid fraction that contains phospholipids, sphingolipids, branched-chain amino acids and oligosaccharides that have been shown to be anti-inflammatory and potentially cardioprotective^(^^1^^)^. Following dairy food processing, the native MFGM structure is disrupted and its components may be found at varying levels in certain dairy products, such as buttermilk or cream, but some proteins derived from MFGM have been found in skimmed milk^(^^22^^)^. Furthermore, the mammalian species the milk is derived from, their diets, and the subsequent levels of different fatty acids may influence the processing dynamics of MFGM^(^^22^^,^^23^^)^.

Individuals are highly variable in terms of diet, genetic composition and metabolic activity and are at different stages of the atherosclerosis continuum, thus making it difficult to come up with established cut-offs to describe postprandial inflammation^(^^24^^)^. Furthermore, clinical markers of lipid metabolism have focused on lipid levels in the fasting state, whereas most of an individual's day is spent in the postprandial state^(^^16^^)^. Numerous studies have measured postprandial inflammation; however, these studies vary in the postprandial blood collection times, the type of markers measured and the fatty acid composition of the test meal^(^^25^^–^^27^^)^. Studies that collected the same markers have shown contrasting results^(^^13^^)^. It is therefore reasonable to argue that postprandial inflammation is not altogether understood. Fortunately, the measurement of postprandial markers of inflammation as opposed to fasting markers is rapidly gaining favour as a means of studying CVD risk, especially the postprandial handling of SFA^(^^28^^,^^29^^)^. Studies have shown that fatty acids derived from dairy products may have a beneficial effect on postprandial inflammation, and that this beneficial effect may stem from the bioactive membrane components of MFGM^(^^20^^)^. Therefore, the present study sought to test whether addition of MFGM to a meal high in saturated fat derived from cream could mitigate the postprandial inflammation experienced in a sample of non-diabetic overweight and obese adults. The present randomised, crossover study measured the plasma inflammatory responses, lipid and metabolic panels and lymphocyte gene expression from baseline to 6 h postprandially.

## Materials and methods

All of the clinical study parameters have been described previously in Demmer *et al*.^(^^13^^)^.

### Participants

A total of thirty-six participants (seventeen men and nineteen women) between 18 and 65 years of age participated in the study. Participants were recruited from Davis, Sacramento, and the surrounding Northern California regions. The inclusion criteria included an overweight BMI (25–29·9 kg/m^2^), and at least two MetS traits according to the American Heart Association (AHA) definition, or an obese BMI (30–39·9 kg/m^2^) and any number of MetS traits. Traits of the MetS include a waist circumference >40 inches (>102 cm) for men and >35 inches (>89 cm) for women; fasting plasma TAG ≥150 mg/dl (≥1·70 mmol/l); fasting plasma HDL-cholesterol <40 mg/dl (<1·04 mmol/l) for men and <50 mg/dl (<1·30 mmol/l) for women; blood pressure ≥130/85 mmHg; and fasting glucose ≥100 mg/dl (≥5·56 mmol/l)^(^^30^^)^. The AHA definition of the MetS includes having at least three of the previous parameters.

Exclusion criteria included diagnosis of an immune-related disease, gastrointestinal disorder, T2DM, eating disorder, allergy to the provided study foods, cancer, pregnancy or lactation, a greater than 10 % change in body weight within the previous 6 months, or poor vein accessibility as evaluated by the study phlebotomist. Additional exclusionary criteria included use of a weight loss medication, daily use of a non-steroidal anti-inflammatory drug, anti-inflammatory supplement, corticoid steroid, tobacco, a change in hormonal birth control use within the previous 6 months, or initiation of statins within the previous 3 months. Additionally, initiation of an exercise programme within the previous 6 months or having plans to become pregnant within 6 months were exclusionary criteria. Dietary exclusionary criteria included consumption of >1 servings of fish per week, >14 g fibre per 1000 kcal (4184 kJ) per d, <16:1 ratio of total dietary *n*-6:*n*-3, >1 % daily energy in the form of *trans*-fats, and a vegetarian diet pattern.

**Fig. 1.**
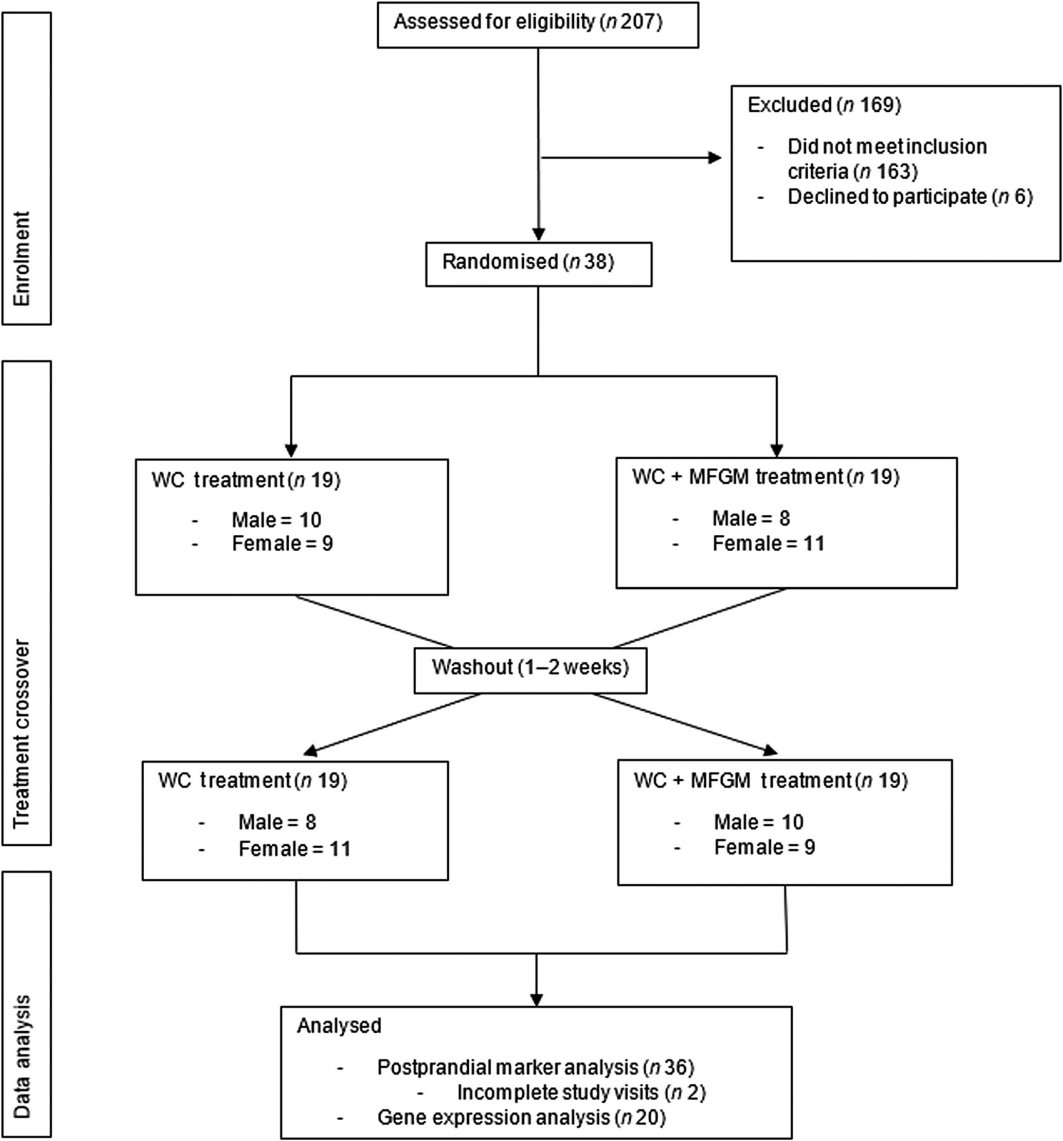
Consolidated Standards of Reporting Trials (CONSORT) diagram of the randomised crossover trial showing enrolment, treatment allocation and analysis of participants. WC, whipping cream; WC+MFGM, whipping cream + milk fat globule membrane.

Eligibility was determined using health history questionnaires that asked potential participants about their health history, diet and medications. During the screening visit, anthropometric measurements (height, weight and waist circumference) were taken, along with a fasting blood sample to evaluate blood lipids and glucose to determine MetS status. Details of recruitment, screening and enrolment can be found in Fig. 1.

This study was conducted according to the guidelines laid down in the Declaration of Helsinki and all procedures involving human subjects/patients were approved by the Institutional Review Board of the University of California, Davis. Written informed consent was obtained from all participants. The study was registered at ClinicalTrials.gov as NCT01811329.

### Study design

Participants were randomised to consume two isoenergetic test meals in a double-blinded, cross-over design. A high-fat whipping cream (WC) meal was compared with a high-fat WC meal with MFGM (WC+MFGM) added. The meals were each consumed in the morning, separated by a 1- to 2-week-long wash-out period to avoid carryover effects. Consumption of anti-inflammatory supplements, non-steroidal anti-inflammatory drugs or alcohol was not permitted for the 72 h preceding the study visit day. Additionally, seafood consumption and vigorous exercise in the 24 h preceding the study visit day were not permitted to avoid confounding changes in inflammatory markers. Compliance was assessed using a 1-d food record representative of the previous 24 h. Analysis of the diet records was performed using the Nutrition Data System for Research (NDSR; University of Minnesota).

Each study visit day, participants arrived at the Western Human Nutrition Research Center following a 10–12 h fast. The participants then completed the 24-h diet record and a modified gastrointestinal questionnaire^(^^31^^)^. The 0 h fasted blood sample was then collected by venepuncture. Anthropometrics, including blood pressure, heart rate, waist circumference and weight were measured. The test meal was consumed within 20 min, and postprandial blood draws were collected at 1, 3 and 6 h postprandially, as determined from previous postprandial clinical trials observing postprandial inflammation following a high-fat test meal^(^^32^^)^. Following the test meal, exercise or consumption of any other food was not permitted for the remainder of the study visit day. Bottled water was allowed, as was leaving the facility by car between time points.

### Dietary challenges

The test meals on both study visit days included either a WC or WC+MFGM smoothie, along with a thin slice of bagel with strawberry preserves. The smoothie contained deionised water, whey protein isolate, raspberry sorbet and anhydrous cream composed of 99·8 % dairy fat^(^^33^^)^. The WC+MFGM smoothie contained the ingredients previously mentioned, as well as 10 % by weight BPC50, a proprietary cream-derived complex milk lipid fraction powder (*β* serum concentrate) supplied by the Fonterra Co-operative Group Ltd (New Zealand). BPC50 contains (% w/w): 52 % protein, which includes 13·2 % membrane-derived, 6·6 % lactose and 36·2 % total fat (22·5 % TAG and 13·7 % phospholipids), 0·63 % gangliosides (GD3) and 5·2 % ash^(^^13^^)^. Fatty acid binding protein, butyrophilin, lactadherin, adipophilin and xanthine oxidase and mucin are the proteins of highest abundance in BPC50. More information on the test meal ingredients can be found in Table 1. Following consumption of the test meal, participants were asked to rinse their smoothie cups and to drink the rinse water.

**Table 1.** Test meal composition (Mean values and standard deviations)

Meal component	WC		WC+MFGM	
	Mean	sd	Mean	sd
Energy				
kcal	1094·2	187·5	1094·2	187·7
kJ	4578·1	784·5	4578·1	785·3
Total fat (g)	68·0	11·6	67·9	11·7
Fat (% total energy)	55·9	0·0	55·9	0·0
Total carbohydrate (g)	83·5	14·3	84·0	14·4
Carbohydrates (% total energy)	30·5	0·0	30·7	0·0
Total protein (g)	42·9	7·3	43·4	7·4
Protein (% total energy)	15·7	0·0	15·9	0·0
Total SFA (g)	41·6	7·1	40·6	7·0
SFA (% total energy)	34·2	0·0	33·4	0·0
Total MUFA (g)	19·4	3·3	19·3	3·3
MUFA (% total energy)	16·0	0·0	15·8	0·0
Total PUFA (g)*	3·2	0·6	3·7	0·6
PUFA (% total energy)*	2·6	0·0	3·0	0·0
SFA 4 : 0 (butyric acid) (% total weight)*	0·4	0·0	0·3	0·0
SFA 6 : 0 (caproic acid) (% total weight)*	0·3	0·0	0·2	0·0
SFA 8 : 0 (caprylic acid) (% total weight)*	0·1	0·0	0·1	0·0
SFA 10 : 0 (capric acid) (% total weight)	0·3	0·0	0·3	0·0
SFA 12 : 0 (lauric acid) (% total weight)	0·4	0·0	0·3	0·0
SFA 14 : 0 (myristic acid) (% total weight)	1·3	0·0	1·1	0·0
SFA 16 : 0 (palmitic acid) (% of total weight)	3·5	0·0	2·9	0·0
SFA 18 : 0 (stearic acid) (% total weight)	1·6	0·0	1·4	0·0
MUFA 16 : 1 (palmitoleic acid) (% total weight)*	0·3	0·0	0·2	0·0
MUFA 18 : 1 (oleic acid) (% of total weight)	3·4	0·0	2·9	0·0
PUFA 18 : 2 (linoleic acid) (% total weight)	0·4	0·0	0·4	0·0
PUFA 18 : 3 (linolenic acid) (% total weight)*	0·2	0·0	0·2	0·0

WC, whipping cream; MFGM, milk fat globule membrane.

* Significant total difference by weight (*P* < 0·05).

The energy content of the meals was formulated to provide 40 % of each participant's daily energy intake, calculated using the National Academy of Sciences equation from the Institute of Medicine Dietary Reference Intake^(^^34^^)^. The Baecke Physical Activity Questionnaire was used to determine physical activity level^(^^35^^)^. The WC and WC+MFGM meals were formulated to vary less than 0·2 % in macronutrients. Each meal provided each participant approximately 55 % (49–87 g) fat, 30 % (61–107 g) carbohydrate and 15 % (31–55 g) protein of total energy intake, scaled to each participant's daily energy need. The addition of MFGM replaced 31 % of the fat in each meal, or 34 % of total energy. The nutrient composition of the test meals was estimated using the NDSR.

### Blood analyses

Whole blood was drawn by venepuncture at baseline (0 h), and at 1, 3 and 6 h postprandially. Red- or gold-top tubes were left to clot at room temperature for 30 min before being centrifuged at 1300 ***g*** at 4°C for 10 min. Whole blood EDTA tubes were placed on ice immediately following the blood draw and were centrifuged within 30 min at 1300 ***g*** at 4°C for 10 min. All serum and plasma tubes were kept on ice during aliquoting, and subsequently placed at −80°C until analysis.

### Inflammatory markers

Inflammatory markers assessed from serum included cytokines (IL-10, IL-1*β*, IL-2, IL-4, IL-6, IL-8, TNF-*α* and monocyte chemoattractant protein-1) and vascular injury molecules (C-reactive protein (CRP), serum amyloid A (SAA), soluble intercellular adhesion molecule and soluble vascular adhesion molecule). The concentration of IL-18 was assessed from plasma. Analyses were performed using a Multi Spot ELISA kit as recommended by the manufacturer, Meso Scale Discovery (SECTOR Imager 2400). Plates came pre-coated with antibodies and were incubated with 25–50 µl of serum or plasma. Labelled detection antibodies were assessed following a washing step. Protein quantification was determined through detection of light emitted by the labelled antibodies upon electrical stimulation. To determine the postprandial inflammatory response, the incremental AUC (iAUC) was calculated for each marker, from 1 h postprandially to 6 h postprandially.

### Metabolic parameters

Whole blood was collected in a 3·5 ml serum-separating gold-top tube, allowed to clot at room temperature and centrifuged at 1300 ***g*** for 20 min. The samples were then assessed for glucose levels and a lipid panel at the baseline, 1 h, 3 h and 6 h time points, and assessed for insulin levels at the baseline, 1 h and 3 h time points. Sample analysis was carried out at the University of California Davis Medical Center Pathology Laboratory.

### Clinical characteristics

Participant height was taken at enrolment using a wall-mounted stadiometer (Ayrton Stadiometer Model S100; Ayrton Corporation). At the beginning of each study visit, body weight and waist circumference were taken in duplicate. Participant body weight was collected using the Calibrates 6002 Wheelchair Scale (Scaletronix). Waist circumference was measured using QM2000 Measure Mate (QuickMedical). A member of the study team measured participant waist circumference midway between the lateral lower rib and the iliac crest while standing. At baseline and at each postprandial time point, blood pressure and heart rate were measured using the GE Instruments Carescape V100 with Critikon Dura-cuff for adults or large adults^(^^13^^)^.

### Lymphocyte isolation

Following centrifugation, the plasma layer was aliquoted and used for inflammatory marker analyses. The lymphocyte layer was isolated and transferred to a tube containing 2 ml PBS. The lymphocyte–PBS solution was carefully transferred to a tube containing Ficoll-Paque before being centrifuged at 450 ***g*** for 10 min. The cells were then resuspended in 1 ml PBS and centrifuged at 1000 ***g*** in a microfuge for 1 min at 4°C. Following centrifugation, the cells were resuspended in 400 µl of RNA*later* (Ambion) and stored overnight at 4°C before being transferred to a −80°C freezer until lymphocyte quantification and RNA extraction were performed.

### Lymphocyte quantification

The lymphocytes were kept frozen until cell counting was performed. An aliquot of the lymphocytes in RNA*later* was 1:10 diluted with PBS pH 7·4 and counted using a haemocytometer under 200× magnification. Following quantification, a volume containing 2 × 10^7^ lymphocyte cells was aliquoted into a 1·8 ml microfuge tube and centrifuged for 30 s using a dome microfuge. The supernatant fraction was removed and the lymphocyte cells were immediately frozen on dry ice prior to RNA extraction.

### RNA extraction

The isolation of total RNA was adapted from the PureLink RNA mini kit (Life Technologies) protocol. A quantity of 1 ml of TRIzol reagent (Ambion) was added to 2 × 10^7^ lymphocytes and vortexed and incubated at room temperature for 5 min. Following the 5-min incubation at room temperature, 0·2 ml chloroform were added, the mixture was vigorously shaken and then centrifuged at 12 000 ***g*** for 15 min at 4°C. Following centrifugation, the aqueous layer was transferred to an RNase-free 1·8 ml microfuge tube and an equal volume of 70 % ethanol was added. After vigorous shaking, the solution was applied on the Spin Cartridge and centrifuged at 12 000 ***g*** for 15 s at room temperature. Once the entire sample was loaded onto the column, 700 µl of Wash Buffer I were added and the mixture was centrifuged at 12 000 ***g*** for 15 s at room temperature. The flow through was discarded and 500 µl of Wash Buffer II were added, followed by another centrifugation step. The sample was centrifuged again to dry the membrane and 30 µl of RNase-free water were added. Following a 1 min-long incubation at room temperature, the column was centrifuged for 2 min and the RNA was transferred to an RNase-free microfuge tube. RNA quality was assessed using an Experion Automated Electrophoresis System (BioRad) and RNA with an RNA quality indicator (RQI) score greater than 9·2 was subsequently used for quantitative PCR analysis.

### TaqMan real-time RT-PCR

The gene expression analysis method has been described previously in Berthelot *et al.*^(^^36^^)^. The High-Capacity cDNA Reverse Transcription kit from Applied Biosystems was used to synthesise first-strand cDNA from 168 ng of total RNA. Baseline (0 h) and 6 h postprandial samples for both WC and WC+MFGM were analysed. Gene expression was quantified in triplicate using a ninety-six-well plate containing four endogenous control genes (actin, glyceraldehyde 3-phosphate dehydrogenase, hypoxanthine phosphoribosyltransferase 1 (*HPRT1*) and glucuronidase *β* (*GUSB*)) as well as custom-designed TaqMan probes and primers. The probes and primers were designed to target ninety-two selected genes involved in inflammation and lipid metabolism. All gene amplification specificities, quality controls and reaction conditions were followed according to Applied Biosystems. Real-time PCR was performed using 50-fold diluted first-strand cDNA and the TaqMan Fast Advanced Master Mix on a Prism 7500 Fast real-time PCR thermocycler, under the following reaction conditions: 50°C/2 min; 95°C/20 s; and forty cycles of 95°C/3 s; 60°C/30 s.

Following amplification, expression levels of the target genes both in baseline samples and postprandial samples were calculated using the Pfaffl method^(^^37^^)^. This was done for each subject and normalised against *HPRT1* and *GUSB*. Amplification efficiencies were analysed for each reaction using LinRegPCR software (Heart Failure Research Center version 2014.2; Academic Medical Center, Amsterdam, the Netherlands). Δ Cycle threshold (ΔCt) values were corrected by their corresponding individual efficiencies.

### Statistical analysis

Participant characteristics, baseline and postprandial markers and gene expression fold changes were all included in the statistical analyses. Sample size was determined using the means and standard deviations of the primary outcome marker, IL-6^(^^38^^)^. Postprandial time points were converted to a single iAUC value to quantify overall dietary response. Order by treatment effects were tested using two-way multivariate ANOVA on all response variables, with subject ID as a random variable. The residuals were then tested for normality using the Shapiro–Wilk test in JMP Pro 13. Normally distributed variables with no treatment by order effects were compared between treatments by testing the hypothesis that the within-subject difference across treatments was equal to zero (paired *t* test). The Wilcoxon signed-rank test was used if the variables were non-normally distributed. Following the paired *t* tests and signed-rank Wilcoxon test, BMI, sex, baseline SAA and baseline CRP were added as additional factors in an adjusted model along with subject and treatment. Secondary analysis using baseline cholesterol:HDL-cholesterol ratio (Chol:HDL) as a covariate was used to test for a treatment effect modification. For all genes with significant effect modification, correlation analyses were performed and *R*^2^ values are reported. The Bonferroni correction was used to correct for the false discovery rate. Statistical significance was set to *P* < 0·05.

## Results

### Participant characteristics

A total of 207 participants were screened for the study, with thirty-eight completing enrolment and being randomised to order 1, in which the WC meal was given on the first test day, or order 2, in which the WC+MFGM meal was given on the first test day. A total of thirty-six participants completed both test days and out of those thirty-six participants, an exploratory convenience subset of twenty was randomly selected for lymphocyte gene expression analysis. The results for the effects of the challenge meals on clinical and inflammatory markers are reported for all thirty-six subjects while the lymphocyte gene expression data are reported for the twenty-subject subset. Approximately half from each order were analysed for lymphocyte gene expression.

Participant characteristics at baseline are shown in Table 2. The baseline parameters were averaged for each participant across the test days to determine order medians. Differences between baseline participant characteristics were not found between study days or between test meals.

**Table 2.** Participant baseline characteristics* and metabolic syndrome (MetS) criteria (Mean values and standard deviations; numbers of participants)

	Mean	sd	MetS criteria
Participants (*n*)			
Male	17		
Female	19		
Age (years)	42·9	13·9	
BMI (kg/m^2^)	31·7	2·6	
Waist circumference (cm)	99·7	7·7	
Waist circumference (males) (cm)	103·9	1·2	>101·6
Waist circumference (females) (cm)	95·3	1·1	>88·9
Systolic blood pressure (mmHg)	124·1	12·9	≥130
Diastolic blood pressure (mmHg)	72·4	10·2	≥85
Chol:HDL†	4·2	1·1	
TAG (mg/dl)‡	132·6	72·6	≥150
Fasting glucose (mg/dl)‡	89·2	13·8	≥100
HDL-cholesterol (all subjects) (mg/dl)‡	49·0	14·9	
HDL-cholesterol (males) (mg/dl)‡	43·1	2·0	<40
HDL-cholesterol (females) (mg/dl)‡	56·4	2·8	<50
CRP (μg/l)	4·2	4·6	
SAA (μg/l)	7·0	23·0	

Chol:HDL, cholesterol:HDL-cholesterol ratio; CRP, C-reactive protein; SAA, serum amyloid A.

* Each value represents a within-subject average from baseline on both study days.

† Chol:HDL >4 represents significantly greater risk for CVD.

‡ To convert TAG from mg/dl to mmol/l, multiply by 0·01129. To convert glucose from mg/dl to mmol/l, multiply by 0·0555. To convert HDL-cholesterol from mg/dl to mmol/l, multiply by 0·02586.

### Test meal composition

The greatest compositional differences between the WC and WC+MFGM meals were in median fatty acid content with total weight of SFA having a 0·93 g difference, PUFA a 0·47 g difference and MUFA a 0·18 g difference. Total PUFA content was significantly higher in the WC+MFGM meal. Linolenic acid was significantly lower in the WC+MFGM meal and arachidonic acid (ARA; 20 : 4) was significantly higher in the WC+MFGM meal. Palmitoleic acid (16 : 1) and linolenic acid (18 : 3) were significantly lower in the WC+MFGM meal. Butyric (4 : 0), caproic (6 : 0) and caprylic (8 : 0) acids were all significantly lower in the WC+MFGM meal.

### Lipid and metabolic parameters

Hourly postprandial clinical data can be found in Tables 3 and 4 for the WC meal and the WC+MFGM meal, respectively.

**Table 3.** Hourly postprandial data for clinical and inflammatory markers following the whipping cream (WC) treatment (Mean values and standard deviations)

Variable	Baseline		1 h		3 h		6 h	
	Mean	sd	Mean	sd	Mean	sd	Mean	sd
IL-10 (pg/ml)	0·52	1·13	0·58	1·15	0·58	1·21	0·55	1·28
IL-1*β* (pg/ml)	0·09	0·18	0·09	0·17	0·08	0·15	0·09	0·18
IL-4 (pg/ml)	0·06	0·05	0·07	0·05	0·06	0·05	0·05	0·05
IL-6 (pg/ml)	0·70	1·10	0·63	1·18	0·60	1·13	0·87	1·51
IL-8 (pg/ml)	11·05	3·44	10·98	3·03	10·09	2·69	10·18	3·06
TNF-*α* (pg/ml)	2·44	0·69	2·38	0·67	2·27	0·57	2·32	0·63
IL-18 (pg/ml)	152·27	88·37	156·90	85·32	162·02	96·78	167·18	92·36
LBP (μg/ml)	3·39	1·30	3·36	1·38	3·26	1·14	3·33	1·21
MCP-1 (pg/ml)	354·88	93·35	358·99	82·68	347·65	88·75	326·69	101·09
CRP (μg/l)	4·23	4·80	4·41	4·88	4·33	4·73	4·35	4·57
SAA (μg/l)	9·34	32·20	8·84	28·77	8·26	25·75	7·78	20·88
sICAM-1 (μg/l)	0·92	0·48	1·00	0·52	0·96	0·49	0·96	0·49
sVCAM-1 (μg/l)	1·49	0·83	1·58	0·90	1·50	0·84	1·53	0·86
Glucose (mg/dl)*	90·47	10·73	80·25	19·30	87·44	11·79	85·81	5·80
Cholesterol (mg/dl)*	202·39	43·47	204·61	39·57	203·42	41·00	206·78	39·94
HDL-cholesterol (mg/dl)*	50·50	14·61	51·14	14·74	49·56	14·33	51·22	14·97
LDL-cholesterol (mg/dl)*	124·31	37·18	114·69	36·06	113·03	38·33	120·69	35·06
Chol:HDL ratio	4·24	1·09	4·26	1·14	4·30	1·29	4·32	1·23
TAG (mg/dl)*	135·08	78·62	193·64	88·41	248·08	138·40	202·97	110·45
Non-HDL-cholesterol (mg/dl)*	151·89	40·09	153·47	37·23	153·86	39·62	155·56	38·31
Insulin (μU/ml)*	15·59	11·09	84·72	68·14	39·94	35·82	−	−

LBP, lipopolysaccharide binding protein; MCP-1, monocyte chemoattractant protein-1; CRP, C-reactive protein; SAA, serum amyloid A; sICAM-1, soluble intercellular adhesion molecule-1; sVCAM-1, soluble vascular cell adhesion molecule-1; Chol:HDL, cholesterol:HDL-cholesterol ratio.

* To convert glucose from mg/dl to mmol/l, multiply by 0·0555. To convert cholesterol from mg/dl to mmol/l, multiply by 0·02586. To convert TAG from mg/dl to mmol/l, multiply by 0·01129. To convert insulin from μU/ml to pmol/l, multiply by 6·945.

**Table 4.** Hourly postprandial data for clinical and inflammatory markers following the whipping cream plus milk fat globule membrane (WC+MFGM) treatment (Mean values and standard deviations)

Variable	Baseline		1 h		3 h		6 h	
	Mean	sd	Mean	sd	Mean	sd	Mean	sd
IL-10 (pg/ml)	0·52	1·08	0·57	1·09	0·57	1·04	0·58	1·17
IL-1β (pg/ml)	0·08	0·10	0·08	0·11	0·07	0·09	0·08	0·10
IL-4 (pg/ml)	0·07	0·05	0·06	0·05	0·07	0·06	0·06	0·06
IL-6 (pg/ml)	0·78	1·43	0·63	1·27	0·60	1·20	0·86	1·48
IL-8 (pg/ml)	11·79	3·64	10·63	4·11	10·40	3·67	10·81	3·17
TNF-α (pg/ml)	2·54	0·83	2·36	0·81	2·32	0·81	2·32	0·73
IL-18 (pg/ml)	154·76	80·18	154·41	78·68	151·73	84·08	156·08	85·30
LBP (μg/ml)	3·32	1·24	3·44	1·26	3·39	1·27	3·51	1·60
MCP-1 (pg/ml)	352·77	94·30	339·34	88·80	309·29	104·27	329·71	86·41
CRP (μg/l)	4·20	4·43	4·39	4·64	4·42	4·79	4·51	4·82
SAA (μg/l)	4·62	5·34	4·79	5·75	4·54	5·36	4·81	5·37
sICAM-1 (μg/l)	0·93	0·50	0·99	0·53	0·97	0·54	0·97	0·51
sVCAM-1 (μg/l)	1·49	0·85	1·56	0·90	1·51	0·87	1·56	0·88
Glucose (mg/dl)*	87·88	16·33	75·06	21·84	88·83	9·83	88·33	7·23
Cholesterol (mg/dl)*	194·61	38·41	199·36	40·45	197·47	38·60	199·14	40·00
HDL-cholesterol (mg/dl)*	47·56	15·35	50·64	16·23	49·14	15·47	50·03	15·64
LDL-cholesterol (mg/dl)*	118·42	34·77	111·81	35·21	100·56	35·27	109·82	35·97
Chol:HDL ratio	4·19	1·15	4·23	1·17	4·32	1·23	4·27	1·17
TAG (mg/dl)*	130·05	67·06	184·44	78·01	243·31	104·38	206·06	106·93
Non-HDL-cholesterol (mg/dl)*	144·72	34·52	148·72	35·91	148·33	35·48	149·08	37·05
Insulin (μU/ml)*	14·38	8·11	52·03	54·43	35·63	24·35	−	−

LBP, lipopolysaccharide binding protein; MCP-1, monocyte chemoattractant protein-1; CRP, C-reactive protein; SAA, serum amyloid A; sICAM-1, soluble intercellular adhesion molecule-1; sVCAM-1, soluble vascular cell adhesion molecule-1; Chol:HDL, cholesterol:HDL-cholesterol ratio.

* To convert glucose from mg/dl to mmol/l, multiply by 0·0555. To convert cholesterol from mg/dl to mmol/l, multiply by 0·02586. To convert TAG from mg/dl to mmol/l, multiply by 0·01129. To convert insulin from μU/ml to pmol/l, multiply by 6·945.

**Fig. 2.**
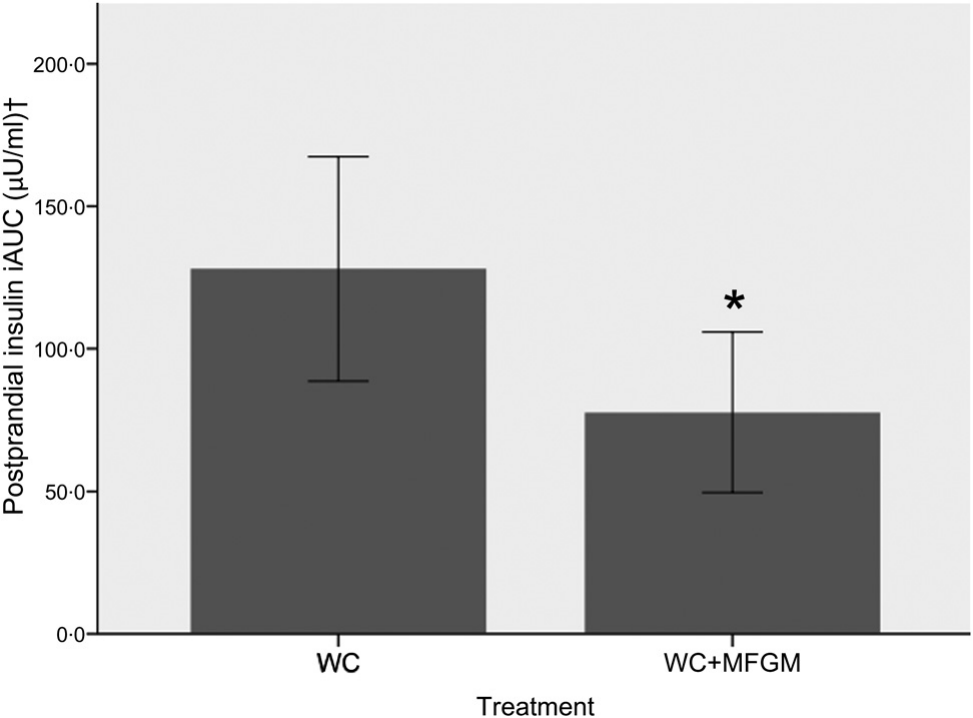
Effect of milk fat globule membrane (MFGM) on insulin response following whipping cream (WC) and WC+MFGM meals. Values are means (*n* 36) of incremental AUC (iAUC) calculated from 1–3 h postprandially, with standard errors represented by vertical bars. * Mean value was significantly different from that for the WC treatment (*P* < 0·01; *P* < 0·05 adjusted). † To convert insulin from μU/ml to pmol/l, multiply by 6·945.

The postprandial insulin iAUC was significantly lower after the WC+MFGM meal as compared with the WC meal (*P* < 0·01) (Fig. 2). Median cortisol iAUC was found to decrease after both meals in the postprandial period, with a greater decrease following the meal with WC alone (*P* = 0·03).

Postprandial TAG, HDL-cholesterol, LDL-cholesterol, Chol:HDL and non-HDL-cholesterol were measured in the postprandial period; however, none was significantly different between test meals. At 3 h postprandially, mean TAG concentration was 248·1 (sd 138·4) mg/dl (2·8 (sd 1·6) mmol/l) after the WC meal and 243·3 (sd 104·4) mg/dl (2·8 (sd 1·2) mmol/l) after the WC+MFGM meal. At 6 h postprandially, mean TAG concentration decreased to 203·0 (sd 110·5) mg/dl (2·3 (sd 1·3) mmol/l) after the WC meal and 206·1 (sd 107·0) mg/dl (2·3 (sd 1·2) mmol/l) after the WC+MFGM meal.

The insulin iAUC response remained statistically significant after adjustment for BMI, sex, baseline SAA and baseline CRP (*P* = 0·04).

### Inflammatory markers

Hourly postprandial inflammatory markers data can be found in Tables 3 and 4 for the WC meal and the WC+MFGM meal, respectively.

**Fig. 3.**
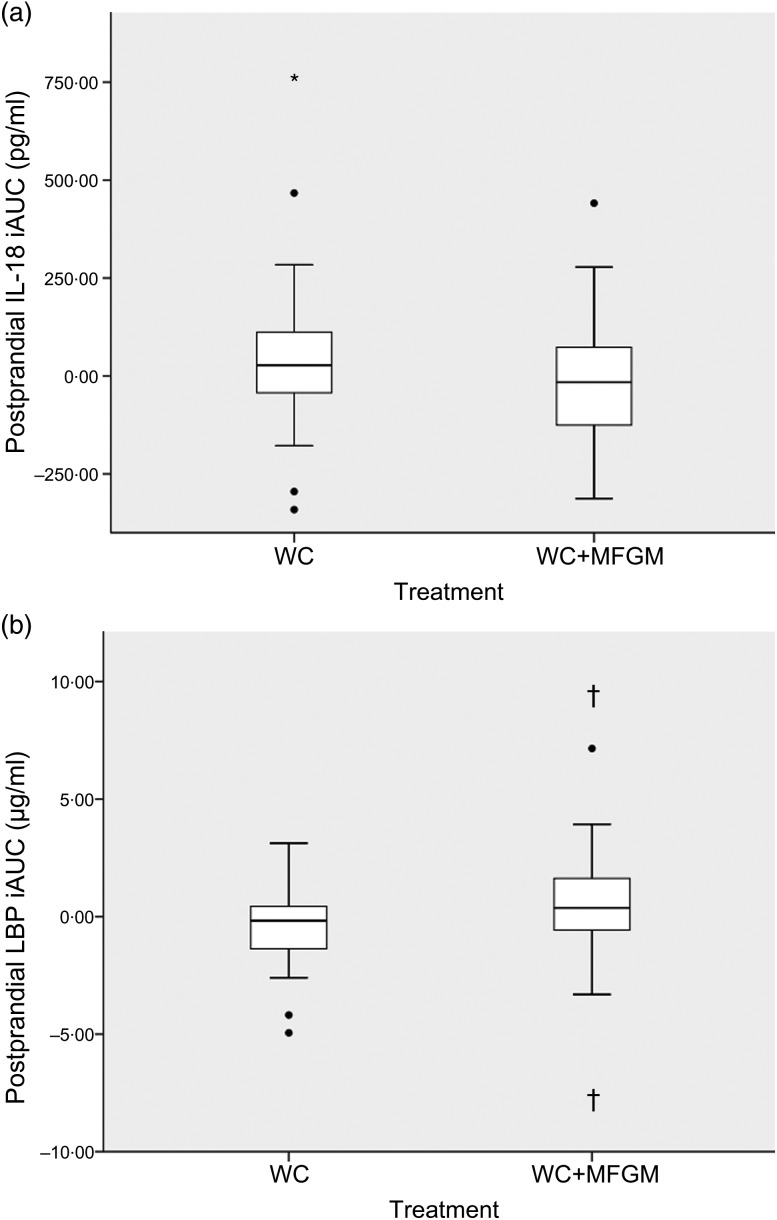
(a) Effect of milk fat globule membrane (MFGM) on IL-8 (a) and lipopolysaccharide binding protein (LBP) (b) responses following whipping cream (WC) and WC+MFGM meals. Values are medians (*n* 36) of incremental AUC (iAUC) calculated from 1, 3 and 6 h postprandially, with ranges represented by vertical bars. * Median value was different from that for the WC treatment (*P* > 0·05 unadjusted; *P* < 0·05 adjusted). † Median value was significantly different from that for the WC treatment (*P* < 0·05 unadjusted; *P* < 0·005 adjusted). ●, Outliers.

IL-18 iAUC was significantly reduced following the WC+MFGM meal after adjustment (*P* = 0·036; Fig. 3(a)). IL-18 was found to reach a peak concentration of 167·2 (sd 92·4) pg/ml at 6 h postprandially following the WC meal. After the WC+MFGM meal, IL-18 reached a peak concentration of 156·1 (sd 85·3) pg/ml at 6 h postprandially; however, this value was nearly the same as the mean baseline concentration. Lipopolysaccharide (LPS) binding protein (LBP) iAUC (Fig. 3(b)) was slightly higher following the WC+MFGM meal (*P* = 0·03, adjusted), reaching its highest concentration of 3·8 µg/ml at 6 h postprandially. Both IL-18 and LBP iAUC levels were not significant before adjusting for BMI, sex, baseline SAA and baseline CRP. IL-18 was not significant between test meals before adjustment (*P* > 0·05), but was significant following adjustment (*P* < 0·05). The outliers pictured are mild outliers and removing them did not change the statistical outcome of the test. The other cytokines and vascular adhesion molecules measured were not significantly different between test meals (IL-10, IL-1*β*, IL-2, IL-4, IL-6, IL-8, TNF-*α*, monocyte chemoattractant protein-1, CRP, SAA and soluble intercellular adhesion molecule).

### Lymphocyte gene expression

**Fig. 4.**
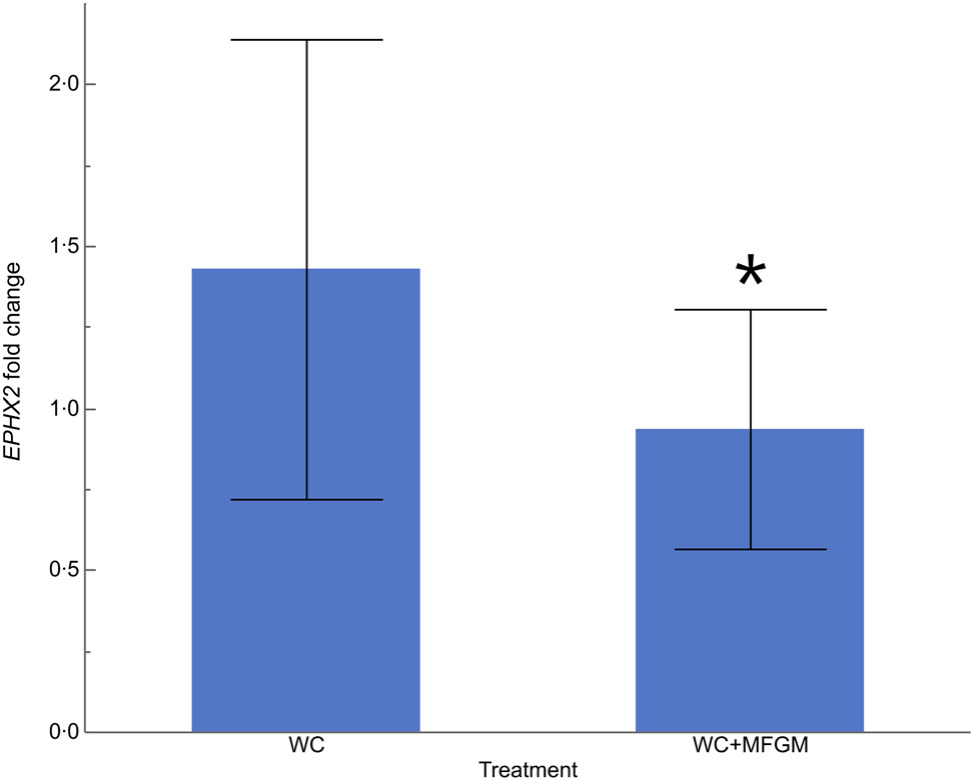
Effect of milk fat globule membrane (MFGM) on fold change of soluble epoxide hydrolase gene (*EPHX2*) in lymphocytes, from baseline to 6 h postprandially. Values are means (*n* 20) of incremental AUC (iAUC), with standard errors represented by vertical bars. * Mean value was significantly different from that for the whipping cream (WC) treatment (*P* < 0·01; *P* < 0·05 after adjustment).

The fold change in gene expression from baseline to 6 h following test meal consumption was measured. Fold change greater than 1·0 indicates increased gene expression, while fold change less than 1·0 indicates reduced gene expression from baseline. Fig. 4 shows that median soluble epoxide hydrolase (*EPHX2*) expression was significantly different following the WC+MFGM meal as compared with the WC meal (*P* = 0·009, *P* = 0·02 after adjustment). The median fold change following the WC meal was 1·23, indicating up-regulation from baseline. Responses across the interquartile range varied from no difference in *EPHX2* expression at 6 h postprandially, to almost 2-fold higher expression at 6 h. After the WC+MFGM meal, the median fold change was approximately 1·0, with participants varying from no change in expression at 6 h, to only 1·3-fold higher expression at 6 h. These fold changes are shown in Table 5. There was a significant treatment by order effect for iAUC percentage eosinophils (*P* = 0·03), and a significant order effect for the iAUC fold change of *CYP4A11* (*P* = 0·03).

**Table 5.** Fold changes in lymphocyte gene expression following whipping cream (WC) and WC + milk fat globule membrane (MFGM) treatments† (Mean values, standard deviations and 95 % confidence intervals; coefficients of determination (*R*^2^))

	WC				WC+MFGM				
Gene	Mean	sd	95 % CI	*R*^2^	Mean	sd	95 % CI	*R*^2^	Baseline Chol:HDL × treatment
*CD14*	1·02	0·36	0·17	0·14	1·07	0·49	0·23	0·46	<0·001*
*LTBR*	1·01	0·43	0·20	0·15	1·08	0·55	0·26	0·29	<0·001*
*BTRC*	1·16	0·44	0·21	0·24	1·16	0·35	0·16	0·28	<0·001
*IRAK1*	1·07	0·26	0·12	0·02	1·02	0·35	0·16	0·51	<0·01
*TLR7*	1·32	0·54	0·25	0·13	1·34	0·44	0·21	0·27	<0·01
*TNFRSF10B*	0·95	0·33	0·15	0·07	1·07	0·46	0·21	0·47	<0·01
*TBXAS1*	0·90	0·43	0·20	0·22	1·11	0·51	0·24	0·25	<0·01
*BCL2*	0·93	0·33	0·15	0·08	1·07	0·48	0·22	0·20	<0·01
*NFKB2*	1·29	0·69	0·32	0·29	1·27	0·51	0·24	0·17	<0·01
*REL*	1·28	0·46	0·21	0·04	1·29	0·65	0·30	0·26	<0·01
*TRAF4*	1·05	0·44	0·21	0·04	1·09	0·31	0·14	0·17	<0·01
*EPHX1*	1·21	0·45	0·21	0·06	1·22	0·59	0·28	0·38	<0·01
*IKBKG*	1·14	0·46	0·21	0·09	1·02	0·45	0·21	0·25	<0·01
*NFKBIE*	1·15	0·48	0·22	0·05	1·07	0·43	0·20	0·16	0·01
*EPHX2*	1·43	0·71	0·33	0·23	0·94	0·37	0·17	0·09	0·01
*TRAF1*	1·27	0·65	0·30	0·19	1·12	0·30	0·14	0·18	0·02
*TLR2*	0·97	0·27	0·12	0·14	1·10	0·46	0·22	0·13	0·02
*TRAF5*	1·22	0·49	0·23	0·09	1·27	0·51	0·24	0·20	0·02
*IRAK1BP1*	1·37	0·69	0·32	0·34	1·41	0·63	0·29	0·06	0·02
*PTAFR*	1·04	0·29	0·14	0·00	0·92	0·34	0·16	0·23	0·03
*BCL3*	1·22	0·48	0·22	0·10	1·02	0·36	0·17	0·10	0·03
*IL1B*	1·06	0·44	0·21	0·16	1·17	0·67	0·31	0·04	0·03
*TNFRSF1A*	1·13	0·49	0·23	0·10	1·05	0·36	0·17	0·18	0·04
*TLR4*	1·07	0·49	0·23	0·13	1·02	0·49	0·23	0·13	0·04
*CSF1*	1·39	0·71	0·33	0·05	1·46	0·69	0·32	0·26	0·04
*PLA2G7*	0·94	0·37	0·17	0·08	1·12	0·60	0·28	0·11	0·04

Chol:HDL, cholesterol:HDL-cholesterol ratio; *CD14*, cluster of differentiation 14; *LTBR*, lymphotoxin β receptor; *BTRC*, *β*-transducin repeat containing E3 ubiquitin protein ligase; *IRAK1*, IL-1 receptor associated kinase 1; *TLR7*, toll-like receptor 7; *TNFRSF10B*, TNF receptor superfamily member 10b; *TBXAS1*, thromboxane A synthase 1; *NFKB2*, NF-κB subunit 2; *TRAF4*, TNF receptor associated factor 4; *EPHX1*, epoxide hydrolase 1; *IKBKG*, inhibitor of NF-κB kinase subunit *γ*; *NFKBIE*, NFKB inhibitor *ε*; *EPHX2*, soluble epoxide hydrolase; *TRAF1*, TNF receptor associated factor 1; *TLR2*, toll-like receptor 2; *TRAF5*, TNF receptor associated factor 5; *IRAK1BP1*, IL-1 receptor associated kinase 1 binding protein 1; *PTAFR*, platelet activating factor receptor; *TNFRSF1A*, TNF receptor superfamily member 1a; *TLR4*, toll-like receptor 4; *CSF1*, colony stimulating factor 1; *PLA2G7*, phospholipase A2 group VII.

* Significant after Bonferroni correction (*P* < 0·05).

† Fold changes after testing for an effect modification of baseline Chol:HDL on treatment. All genes listed were significant for the effect modification.

### Effect of baseline markers on gene expression following milk fat globule membrane treatment

**Fig. 5.**
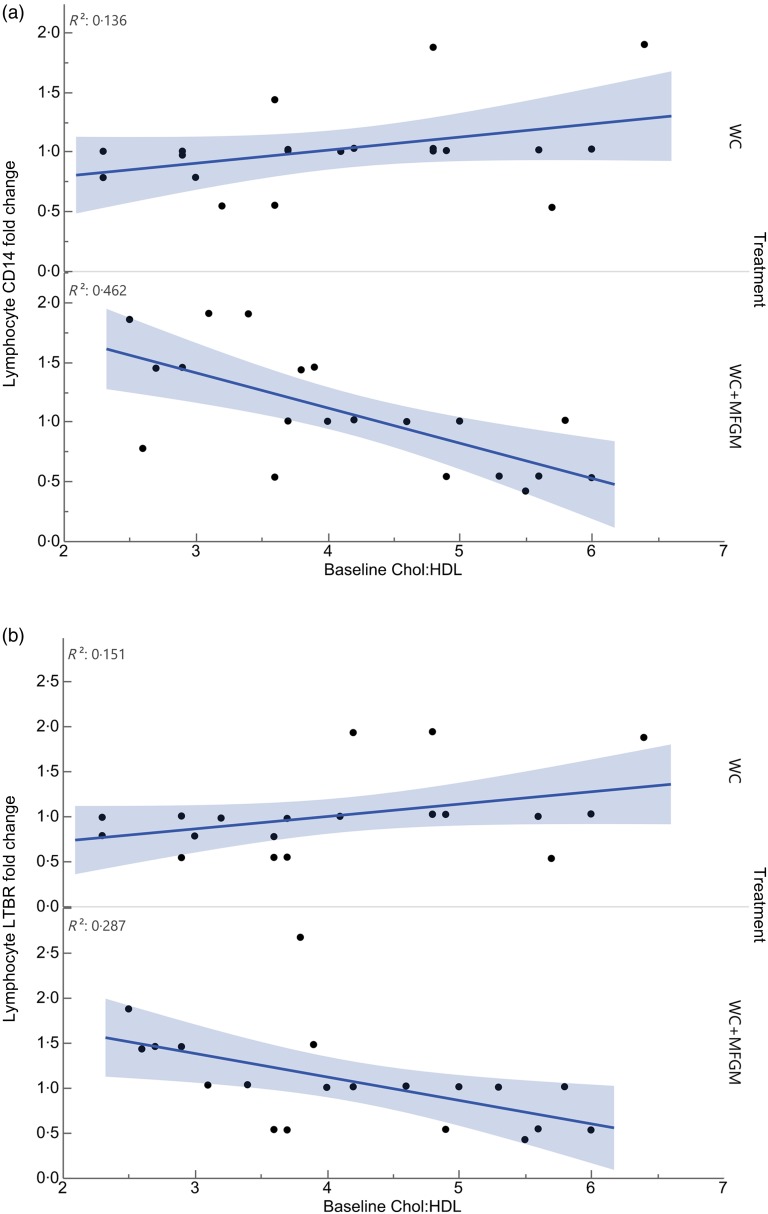
Effect modification of baseline cholesterol:HDL-cholesterol (Chol:HDL) on (a) lymphocyte cluster of differentiation 14 (*CD14*) and (b) lymphotoxin *β* receptor (*LTBR*) gene expression fold changes from baseline to 6 h postprandially (*n* 20) (*P* < 0·001). WC, whipping cream; WC+MFGM, whipping cream + milk fat globule membrane.

Lymphocyte gene expression following MFGM treatment showed significant differences based on baseline Chol:HDL. The fold changes of the following genes were significantly different when considering the interaction between baseline Chol:HDL and treatment: cluster of differentiation 14 (*CD14*), lymphotoxin β receptor (*LTBR*), *BTRC*, *IRAK1*, *TLR7*, *TNFRSF10B*, *TBXAS1*, *BCL2*, *NFKB2*, *REL*, *TRAF4*, *EPHX1*, *IKBKG*, *NFKBIE*, *EPHX2*, *TRAF1*, *TLR2*, *TRAF5*, *IRAK1BP1*, *PTAFR*, *BCL3*, *IL1B*, *TNFRSF1A*, *TLR4*, *CSF1* and *PLA2G7* (Table 5). Following Bonferroni correction, only differences in *CD14* and *LTBR* expression remained significant. The relationship between Chol:HDL, treatment, and both *CD14* and *LTBR* fold changes are shown in Fig. 5. Individuals with a higher Chol:HDL at baseline were more likely to show decreased *CD14* expression with the addition of MFGM. In the absence of MFGM, individuals with higher baseline Chol:HDL were more likely to show no change or slightly increased *CD14* expression. For *LTBR*, the addition of MFGM also had a stronger effect on individuals with a higher baseline Chol:HDL.

## Discussion

The addition of MFGM to a high-SFA dairy product-based meal resulted in a significant decrease in insulin response and attenuation of lymphocyte soluble epoxide hydrolase (sEH) expression. Secondary analyses showed that the effect of MFGM on the expression of *CD14* and *LTBR* is modified by fasting baseline Chol:HDL. These findings suggest that adding MFGM to a meal high in dairy fat beneficially decreases postprandial insulinaemia and sEH induction in overweight and obese individuals, and would be particularly beneficial for those individuals with a high Chol:HDL.

Native MFGM consists of a phospholipid trilayer which arises from the endoplasmic reticulum and plasma membrane in the mammary epithelial cell. The structure allows for the TAG-rich fraction of milk to be separated from the water-soluble fraction^(^^39^^)^. MFGM carries a wide array of lipids and proteins, including phospholipids, glycolipids and sphingolipids. These components are not only important to the bactericidal properties of MFGM through their ability to form lipid rafts, but also deliver these lipids for distribution in cells of the body^(^^23^^,^^39^^)^. Milk products that have undergone various levels of processing and homogenisation retain some of the components of MFGM but not in the native structure^(^^22^^)^. This study was not designed to test which specific components are directly responsible for the observed effects.

Postprandial insulin was found to be significantly reduced following consumption of MFGM. This response was significant when looking at all participants regardless of baseline characteristics or MetS traits. This insulin response was consistent with another experiment in the same subject population testing the effects of adding MFGM to a meal high in palm oil^(^^13^^)^. This previous study by Demmer *et al*.^(^^13^^)^ showed that addition of MFGM to palm oil significantly reduces insulin by 50 %, with a corresponding decrease in postprandial glucose concentration, a significant increase in TAG, and a decrease in LDL-cholesterol. The present study found no difference in postprandial glucose concentration between WC and WC+MFGM test meals, suggesting that for glucose, the effect of MFGM may depend on whether it is consumed with dairy or palm oil, whereas a similar effect was seen for insulin and TAG response despite the source of the saturated fat. Additionally, the study by Demmer *et al*.^(^^13^^)^ demonstrated a significant effect modification of baseline CRP concentration on postprandial IL-6 response following the palm oil meal. Therefore, the present analysis included baseline CRP in the adjusted model.

Neither changes to cholesterol levels nor TAG were apparent within the 6 h postprandial collection window in the present study. A study by Irawati *et al*.^(^^14^^)^ investigated the postprandial response of healthy normal subjects following a high-fat meal (40 g fat from a mixed meal containing either palm oil, coconut oil or rice bran oil). They found that across all treatments ten out of twenty-six participants show 4 h postprandial TAG levels greater than 1·7 mmol/l (approximately 150 mg/dl). These TAG levels are not related to fasting cholesterol levels or even fasting TAG. The results from Irawati *et al*.^(^^14^^)^ suggest that fasting lipids may not be an appropriate indicator of how the body handles postprandial TAG. Another study by Schmid *et al*.^(^^21^^)^ looked at blood lipids following high-fat meals either with or without dairy products. They fed healthy men a meal in which 60 % of the energy came from fat. At 4 h postprandially, they found TAG iAUC significantly increased following the high-fat dairy product meal. They also found that the meal containing dairy fat resulted in no significant increases in the inflammatory cytokines IL-6, TNF-*α* and CRP, compared with the meals without dairy fat^(^^21^^)^. This suggests that dairy product-derived fat is not more inflammatory than palm oil-derived saturated fat. The increased TAG levels following the WC+MFGM meal in this study may be related to the significantly lower magnitude of the postprandial insulin rise that was also observed after the WC+MFGM meal. Insulin is a hormonal regulator of lipoprotein lipase, which converts TAG into glycerol and NEFA that diffuse into the adipose tissue before being reassembled into TAG for storage^(^^40^^)^. A lower rise in insulin would suggest lowered lipoprotein lipase activity, allowing more TAG to remain in the plasma. However, to fully appreciate the contributing factors to the observed TAG results, hepatic uptake and release should also be assessed. Another possibility is that peripheral glucose uptake is favoured in the postprandial state following MFGM treatment. After the WC+MFGM meal, mean 1 h plasma glucose was 75·1 mg/dl (4·2 mmol/l), slightly lower than the 80·3 mg/dl (4·5 mmol/l) plasma glucose following WC alone, but not statistically significant. Future studies should examine these possibilities to determine whether the overall net effect of MFGM is beneficial on postprandial glucose metabolism and lipaemia.

IL-18 was found to decrease and LBP to increase following the addition of MFGM; however, these differences did not reach significance until after adjusting for BMI, sex, baseline SAA and baseline CRP. IL-18 is a pro-inflammatory cytokine linked to CVD risk and is related to the production of other pro-inflammatory cytokines such as TNF-*α* and IL-1β^(^^13^^)^. Esposito *et al*.^(^^41^^)^ have shown that IL-18 increases following acute hyperglycaemia. Increased IL-18 can stimulate macrophage differentiation and may induce expression of adhesion molecules^(^^41^^)^. Future studies should address whether MFGM reduces IL-18 expression in order to determine if MFGM is beneficial in the postprandial state.

LBP binds free plasma LPS and can stimulate macrophages to produce soluble CD14, which then can facilitate transfer of LPS to HDL, allowing clearance through reverse cholesterol transport activity^(^^42^^)^. A study by Laugerette *et al*.^(^^43^^)^ found that in healthy males, chronic overfeeding results in a higher plasma LBP level after the intervention. It was also noted that participants with a higher fasting LBP level before the study intervention had a higher LBP:soluble CD14 ratio at the end of the study^(^^43^^)^. LBP had the highest range of responses at 6 h postprandially, with responses from 1·4 to 9·3 µg/ml. These findings underscore the importance of studying the interindividual variability in inflammatory response following different meals and suggest that some individuals are particularly susceptible to the pro-inflammatory effects of certain types of meals.

Lymphocyte expression of *EPHX2* was attenuated with addition of MFGM. *EPHX2* encodes sEH, an enzyme expressed in many tissues including the heart, kidneys, liver and vascular endothelium^(^^44^^)^. sEH converts the epoxyeicosatrienoic acids formed from cytochrome P450 metabolism of fatty acids such as ARA, DHA, *α*-linolenic acid (ALA) and linoleic acid (LA) into their corresponding diols such as dihydroxyeicosatrienoic acids^(^^45^^)^. Whereas this conversion allows for increased solubility, lower bioactivity and better excretion of the epoxyeicosatrienoic acid products of ARA, the dihydroxyeicosatrienoic acids have been shown to have proinflammatory activity^(^^45^^,^^46^^)^. In the absence of sEH, epoxyeicosatrienoic acids exert their effects for a longer period of time. The epoxides and diols produced from different precursor fatty acids (DHA, ALA, LA, etc.) have slightly different biological activities compared with the ARA-derived metabolites^(^^45^^)^. Epoxyeicosatrienoic acids produced from ARA are resolvers of inflammation, play a role in lowering pain and blood pressure, and can influence the production of and tissue responsiveness to insulin^(^^47^^)^. Although the present study found no difference in glucose levels, insulin levels were significantly reduced with the MFGM meal. Although the link between MFGM, sEH and insulin metabolism is not yet known, the present study provides additional evidence of an effect of MFGM on insulin signalling pathways as well as pathways involved in the resolution of inflammation.

Baseline Chol:HDL was analysed to test for an effect modification on treatment. Chol:HDL has been reported to be a predictor of not only CVD, but T2DM as well^(^^48^^,^^49^^)^. Several large prospective studies have shown that the higher the Chol:HDL, the more likely individuals will experience a CVD event, with a ratio of 5·5 presenting moderate atherogenic risk^(^^48^^)^.

All genes that were affected by baseline Chol:HDL under the MFGM treatment showed either a decrease or an attenuation of fold change from baseline to 6 h postprandially as the baseline Chol:HDL increased, suggesting that MFGM may be particularly beneficial in individuals with high-risk lipoprotein profiles (Table 5). After correcting for the false discovery rate, only *CD14* and *LTBR* remained significant. Lymphocyte *CD14* fold change showed a slight elevation under the WC meal alone with increasing baseline Chol:HDL. Upon addition of MFGM, the participants with higher baseline Chol:HDL had the greatest reductions in lymphocyte *CD14* gene expression. Under the MFGM test meal, five out of seven participants with reductions in *CD14* gene expression had baseline Chol:HDL ratios greater than 4·5. The same trend was seen for *LTBR*, in that the participants with the highest Chol:HDL ratios at baseline experienced the most significant reduction in *LTBR* fold change. When these high Chol:HDL ratio participants were given the WC meal, they generally had *LTBR* fold change increases. This suggests that MFGM plays a protective role for those individuals at a higher atherogenic risk based on Chol:HDL. One role of CD14 is to facilitate the removal of LPS from plasma^(^^50^^)^. Baseline Chol:HDL had no effect modification on plasma LBP response (*P* = 0·73) directly; however, addition of MFGM to the high-fat meal increased postprandial LBP. Both CD14 and LBP can carry or exchange LPS in the plasma. Converting the plasma LBP response into a baseline to 6 h fold change showed slight increases from baseline following both meals, whereas lymphocyte CD14 fold changes were smaller following the WC+MFGM meal. LTBR is primarily expressed on the vascular endothelium and hepatocytes; however, it is also expressed on monocytes^(^^51^^)^. LTBR binds lymphotoxin ligands on lymphocytes and is involved in inflammatory signal transduction pathways as part of the TNF family of receptors^(^^51^^)^. Although the role of LTBR in CVD is not completely clear, recent literature suggests that it is associated with the promotion of atherosclerosis^(^^51^^,^^52^^)^. Therefore, the results from the present study indicate a protective role of adding MFGM to dairy fat for those individuals with higher baseline Chol:HDL.

The present study was designed to test the effects of adding MFGM to a high-fat meal to understand whether adding back complex lipid and protein components that would normally be present in unpasteurised, unhomogenised milk would attenuate postprandial inflammation. This study provides several investigative directions for future postprandial studies. The MetS population is highly varied, with different symptoms resulting from different underlying metabolic processes. It will therefore be necessary to study clinically similar cohorts to come up with specific MFGM recommendations in future studies.

### Conclusion

The present study shows that addition of MFGM to a meal high in saturated fat can have significant effects on postprandial inflammation and metabolism. The results here warrant future research on how MFGM as a dietary additive can be beneficial to those individuals whose lifestyle, diet and genetics predisposes them to chronic inflammation, insulin resistance and CVD. Treatment strategies encompassing diet, medication, lifestyle changes and incorporation of nutraceuticals like MFGM may accelerate the redevelopment of healthy metabolic profiles in these individuals.
